# Evaluation of Biofilm Inhibitory Activity of Probiotics and Postbiotics Using In Vitro Biofilm Model of Canine Periodontal Disease

**DOI:** 10.3390/microorganisms13112472

**Published:** 2025-10-29

**Authors:** Achraf Adib Lesaux, Eva Cunha, Nathalie Ballet, Manuela Oliveira

**Affiliations:** 1 Phileo by Lesaffre, 59700 Marcq-en-Baroeul, France; 2 CIISA—Centre for Interdisciplinary Research in Animal Health, Faculty of Veterinary Medicine, University of Lisbon, Avenida da Universidade Técnica, 1300-477 Lisbon, Portugal; evacunha@fmv.ulisboa.pt (E.C.); moliveira@fmv.ulisboa.pt (M.O.); 3 Associate Laboratory for Animal and Veterinary Science (Al4AnimalS), Av. da Universidade Técnica, 1300-477 Lisbon, Portugal; 4 Lesaffre International, 59700 Marcq-en-Baroeul, France; n.ballet@lesaffre.com; 5 cE3c—Centre for Ecology, Evolution and Environmental Changes & CHANGE—Global Change and Sustainability Institute, Faculty of Sciences, University of Lisbon, Campo Grande, 1749-016 Lisbon, Portugal

**Keywords:** polymicrobial biofilm, dogs, periodontal disease, postbiotic

## Abstract

Periodontal disease (PD) is one of the most widespread oral inflammatory diseases in dogs, with the potential to cause systemic consequences. The purpose of this study was to evaluate the inhibitory potential of yeast-derived postbiotics and probiotic bacterial strains by using a previously developed in vitro biofilm model mimicking canine PD-associated biofilm including five bacterial species: *Neisseria zoodegmatis*, *Corynebacterium canis*, *Porphyromonas cangingivalis*, *Peptostreptococcus canis*, and *Enterococcus faecalis*. After we confirmed the presence of these five bacterial species by employing Fluorescence In Situ Hybridization, the biofilm inhibitory and eradication activity of 11 yeast-derived postbiotics and probiotic bacterial strains, as well as selected dual biotic combinations, against the polymicrobial biofilm were determined using a modified version of the Calgary Biofilm Pin Lid Device and the crystal violet method; additionally, hemolytic activity was evaluated using canine red blood cells. The results show that the inhibitory activity against the polymicrobial PD biofilm ranged from 0% to 22.55%, and eradication ability varied between 0% and 17.28%; however, when combined, the biotics achieved a maximum inhibition rate of 71%. Probiotic strain BC-05 exhibited the lowest in vitro hemolytic activity. Overall, based on the results, four yeast-derived postbiotics and one probiotic bacterial strain were selected as promising candidates for further evaluation, aiming at in vivo application.

## 1. Introduction

Periodontal disease (PD) is one of the most widespread oral inflammatory diseases in dogs. Previous studies reported PD prevalences higher than 80% in dogs over two years of age, reaching 100% in some breeds [1,2]. PD is initiated by the formation of a pellicle constituted by a saliva-derived layer of glycoproteins that coats the surface of the tooth; this is followed by bacterial adherence to this layer, which results in the formation of polymicrobial biofilm, also known as dental plaque [3]. Dental plaque microbiota is highly complex and distinct according to the PD stage [4]. Moreover, dysbiosis of the oral microbiome, characterized by an imbalance between commensal and pathogenic bacteria, can trigger inflammation, tissue destruction, and PD progression [4,5]. Specific bacterial species, including *Porphyromonas gingivalis*, *Tannerella forsythia*, and *Treponema denticola*, have been associated with PD development; they disrupt host immune responses, promote biofilm formation, and release virulence factors that exacerbate tissue damage [3,5]. These bacteria can infiltrate the subgingival space and produce several metabolites, including ammonia and volatile sulfur compounds, which lead to halitosis, as well as bacterial endotoxins and proteolytic enzymes, promoting periodontal inflammation and stimulating the animal’s immune system [3]. Persistent host inflammation leads to PD progression, which can cause several local and systemic consequences [6].

The treatment of PD requires diagnosis by a veterinarian and may involve professional cleaning and appropriate home care [7]. However, consistent home oral hygiene practices such as toothbrushing can be challenging for pets and owners. Although the chemicals currently used, such as chlorhexidine gluconate, offer immediate benefit regarding PD progression, they may also present some adverse effects, such as gastric disorders, dysgeusia, mucosal irritation, and tooth discoloration, which can impair continued use [8].

New preventive and therapeutic approaches to controlling PD in companion animals are required. Probiotics and postbiotics have emerged as promising candidates for modulating the oral microbiome [9]. By promoting the proliferation of beneficial bacteria and inhibiting that of pathogenic species, these biotics can reduce inflammation, prevent biofilm formation, and improve oral health [9,10,11]. Moreover, oral biotics may reach the gastrointestinal tract, influence the gut microbiome, and exert systemic effects. In fact, there is a close connection between the oral and gut microbiomes, indicating that the modulation of the oral microbiota may have broader health implications [12].

The goal of this study was to evaluate the inhibitory potential of 11 biotics, including eight yeast-derived postbiotics and three probiotic bacterial strains, using an in vitro biofilm model mimicking canine PD-associated biofilm which included bacterial species involved in the different stages of PD in dogs.

## 2. Materials and Methods

### 2.1. Biofilm Bacterial Collection

The 5 bacterial strains used in this study were selected due to their association with different stages of PD progression: *Neisseria zoodegmatis* CCUG 52598T (from a human wound following a dog bite), *Corynebacterium canis* CCUG 58627T (from a human wound following a dog bite), *Peptostreptococcus canis* CCUG 57081 (from canine dental plaque), *Porphyromonas cangingivalis* DSMZ VPB 4874 (from a canine periodontal pocket), and an *Enterococcus faecalis* isolate from a collection of oral bacteria obtained from dogs with PD [13,14].

### 2.2. Culture Conditions

Each bacterial species was propagated according to its specific growth requirements. *N. zoodegmatis* CCUG 52598T, *C. canis* CCUG 58627T, and *E. faecalis* were grown on Brain Heart Infusion (BHI) agar plates (VWR, Leuven, Belgium) under aerobic conditions for 24 h at 37 °C; *P. canis* CCUG 57081 on Chocolate Agar plates (VWR, Leuven, Belgium) under anaerobic conditions for 48 h at 37 °C; and *P. cangingivalis* DSMZ VPB 4874 on Columbia Blood agar plates (VWR, Leuven, Belgium) under anaerobic conditions for 48 h at 37 °C. All bacteria were also grown on *Brucella* Broth medium (Liofilchem, Roseto degli Abruzzi, Italy), supplemented with hemin (5 µg/mL) (Sigma-Aldrich, St. Louis, MO, USA) and vitamin K1 (1 μg/mL) (Liofilchem, Roseto degli Abruzzi, Italy) [14]. After propagation, the purity of each bacterial culture was verified based on macroscopic observation and microscopic examination using Gram staining.

### 2.3. Preparation of Yeast-Derived Postbiotics and Probiotic Bacterial Strains

A total of 8 yeast-derived postbiotics (YE-2, YE-3, YE-4, YE-5, YE-6, YE-7, YCW-1, and YCW-2) and 3 probiotic bacterial strains (BC-23, BC-05, and BC-37) (Lesaffre, Marcq-en-Baroeul, France) were tested in this study. A stock suspension of each postbiotic was prepared in distilled sterile water (see Table 1) by diluting it to each specific concentration according to the experiment: in the spot-on-lawn technique, the concentrations used were 50%, 20%, and 10%; in the experiments evaluating the antimicrobial activity of the postbiotics, conducted using 96-well microplates, the stock suspension of each ingredient was subjected to two-fold serial dilutions ten times. The probiotic bacterial strains were propagated using BHI agar plates and suspended in 0.9% NaCl to achieve 10^9^ CFU/mL suspensions.

### 2.4. Preparation of Canine Artificial Saliva

A suspension of canine artificial saliva (CAS) was prepared, as described by Sanguansermsri et al. [5], using Lab Lemco Powder (Thermo Fisher Scientific, Roskilde, Denmark), yeast extract (Sigma-Aldrich, St. Louis, MO, USA), proteose peptone (Merk, Darmstadt, Germany), hog gastric mucin (Sigma-Aldrich, USA), NaCl, KCl, CaCl_2_, and urea (Alfa Aesar, Kandel, Germany).

### 2.5. Collection of Saliva from Healthy Dogs

After the owner’s consent was obtained, saliva samples were collected from voluntary healthy dogs by using a specific container (Salivette^®^, Sarstedt, Nümbrecht, Germany). Then, the collected saliva was centrifuged (10 min at 5000 rpm), filtered using a 0.22 μm membrane filter, and kept at −20 °C until further use [15,16].

### 2.6. Preliminary Screening of Inhibitory Activity of Yeast-Derived Postbiotics and Probiotic Bacterial Strains

The initial antimicrobial screening of the postbiotic compounds and probiotic microbial strains was performed using a spot-on-lawn technique [15,16]. To this end, each of the bacterial species under study (*N. zoodegmatis* CCUG 52598T, *C. canis* CCUG 58627T, *P. cangingivalis* DSMZ VPB 4874, *P. canis* CCUG 57081, and the clinical *E. faecalis* isolate) was evaluated individually. First, bacterial suspensions at 3 different concentrations, namely, 10^6^ CFU/mL, 10^7^ CFU/mL, and 10^8^ CFU/mL, were prepared in 0.9% NaCl, except for *C. canis*, for which the 10^6^ CFU/mL suspension was not tested. Each suspension was evenly spread on a plate with species-specific culture medium in order to form a bacterial lawn. Then, 10 µL of the different concentrations of each postbiotic was spotted on the plate, followed by incubation at 37 °C under the previously mentioned species-specific culture conditions. Three concentrations of extracts were tested (10%, 20%, and 50%), except for YCW-1, which was tested at 10% and 20%, and YCW-2, which was tested at 10%. In the case of the probiotic bacterial strains, a 10^9^ CFU/mL suspension of each was used. At the end of the incubation period, the antimicrobial potential of each biotic was assessed based on the observation of an inhibition halo around the its spot by using three categories: complete inhibition, observed as a clear inhibitory halo around the spot; incomplete inhibition, when an inhibitory halo was present but not well defined, with a reduction in bacterial growth; and no inhibition, when no halo was detected and a homogeneous bacterial lawn was observed. All experiments were performed in triplicate on separate days.

### 2.7. Production of Polymicrobial Biofilm

A polymicrobial biofilm composed of the five bacterial species previously mentioned was developed based on the protocol described in [14]. To this end, a peg lid (Nunc™ Immuno TSP Lids, Thermo Scientific™, Waltham, MA, USA), previously incubated at 37 °C for 2 h in CAS, was applied to a 96-well microplate (Nunc™, Thermo Scientific, Waltham, MA, USA), filled with 40 μL of suspension of each bacterial species in supplemented *Brucella* broth at a concentration of 10^7^ CFU/mL. The microplate was then subjected to a microaerophilic environment (Merk, Darmstadt, Germany) and incubated for 48 h at 37 °C to enable biofilm establishment on the pegs.

### 2.8. Biofilm Evaluation Based on Fluorescence In Situ Hybridization (FISH)

The biofilm produced was evaluated with FISH, using a protocol previously described by Cunha et al. (2020) [14]. First, ten-well Teflon slides (Heinz Herenz, Hamburg, Germany) were treated and used as hybridization supports [17]. Next, 10 µL of the bacterial suspensions formed by the polymicrobial biofilm previously obtained was pipetted onto the wells of the Teflon slide, allowed to air-dry for fixation, and incubated with 4% paraformaldehyde (*w*/*v*) in PBS for 4 h at room temperature. Afterwards, dehydration was performed by separately incubating the slides with ethanol at 50, 80, and 96% concentrations for 3 min; this was followed by bacterial permeabilization with lysozyme (0.5 µg/mL) (Sigma-Aldrich, St. Louis, MO, USA), incubation for 20 min at room temperature, and then a second dehydration step. Next, 10 µL of hybridization buffer (composed of 0.9 M NaCl, 20 mM Tris–HCl at pH 7.2, 0.01% SDS, and 5 ng/mL of each specific probe (STABVIDA, Caparica, Portugal) reported in Table 2) was added to each well. The slides were incubated for 3 h in a humid chamber (Omnislide Thermal Cycling Block, Hybaid Omnislide System, Thermoelectron Corporation, Waltham, MA, USA) at different temperatures depending on the targeted bacterial species: at 46 °C for *N. zoodegmatis*, *E. faecalis*, and *P. cangingivalis* detection; at 48 °C for *C. canis* detection; and at 35 °C for *P. canis* detection. Then, the slides were incubated in a washing buffer (composed of 0.9 M NaCl, 20 mM Tris–HCl at pH 7.2, and 0.1% SDS) for 15 min at the temperatures previously mentioned. Finally, the slides were mounted in Vectashield Mounting Medium (Vector Laboratories, Newark, CA, USA) and visualized using fluorescent microscopy at 1000X (HCX PLAN APD objective) with a Leica microscope (Leica Microsystems Lda., Lisbon, Portugal) equipped with a 100W mercury lamp, an I3 filter for excitation between 450 and 490 nm, and an N2.1 filter for excitation between 515 and 560 nm.

### 2.9. Determination of Minimum Biofilm Inhibitory Concentration (MBIC) and Minimum Biofilm Eradication Concentration (MBEC) of Yeast-Derived Postbiotics and Probiotic Bacterial Strains Using Crystal Violet Method and FISH

Polymicrobial biofilm was obtained as previously described, and its susceptibility to the yeast-derived postbiotics was determined using a modified version of the Calgary Biofilm Pin Lid Device [14]. After biofilm formation, the peg lid was washed three times in 0.9% NaCl and transferred to a new microplate filled with 100 µL of fresh *Brucella* broth and 100 µL of suspension of each postbiotic at two-fold-reduced concentrations, where the final concentration range tested for YE was from 50% to 0.1%; for YCW-1, from 12.5% to 0.02%; and for YCW-2, from 5% to 0.01%. Next, microplates were incubated for 24 h at 37 °C under microaerophilic conditions, and the MBIC value was determined using crystal violet staining as described in [23,24]. Afterwards, for MBEC determination, the peg lid was washed three times in 0.9% NaCl and transferred to a 96-well plate containing 200 μL of freshly supplemented *Brucella* broth. The plate was sealed and incubated in an ultrasonic bath for 15 min at high frequency (50–60 Hz), after which the peg lid was replaced with a conventional lid. Next, the microplate was incubated under microaerophilic conditions for 48 h at 37 °C, and the MBEC value was determined by optical density measurement at 600 nm (OD600) using a microtiter plate reader (BMG Labtech, FLUOstar OPTIMA, Ortenberg, Germany). A positive control (biofilm bacterial suspensions without treatment) and a negative control (medium only) were included in all assays.

In addition, the suspensions from the wells containing the positive controls and those from the wells in which higher inhibition and eradication percentages were observed were collected and evaluated using FISH to determine bacterial species composition. This evaluation enabled us to conduct a qualitative analysis of the reduction in each bacterial species after its exposure to the action of each biotic based on a direct comparison with the positive control. The evaluation of the bacterial quantity in each sample was performed based on a scale of 1 to 5, where 3 corresponds to the bacterial quantity observed in the positive control of each replicate; thus, the scores of 1 and 2 correspond to a bacterial quantity relatively lower than the one present in the positive control suspension and 4 and 5 to a relatively higher quantity (Table 3). All experiments were conducted in triplicate on separate days.

### 2.10. Inhibition and Eradication Potential of Dual Combinations of Yeast-Derived Postbiotics and Probiotic Bacterial Strains

Based on the individual evaluation results, the postbiotics and probiotics that showed higher inhibitory activity were selected for the evaluation of the inhibition and eradication potential of their dual combinations (1:1; see Table 4) against the polymicrobial biofilm as previously described in Section 2.9. Experiments were performed in triplicate on separate days.

### 2.11. Inhibitory Activity of Postbiotics and Probiotics in the Presence of Canine Saliva

To determine the potential influence of canine saliva on the inhibitory activity of the postbiotics and probiotics, the spot-on-lawn procedure was performed as previously described in Section 2.6. To this end, individual bacterial suspensions of each of the five bacterial species under study at 10^8^ CFU/mL were inoculated on top of the agar medium to produce bacterial lawns. The biotics with higher inhibitory activity, selected based on the results of previous assays, were prepared as stock suspensions (see Table 1) and diluted in canine saliva in a ratio of 1:1 to perform the spot-on-lawn assay. Experiments were performed in triplicate on separate days.

### 2.12. Evaluation of Hemolytic Potential of Yeast-Derived Postbiotics and Probiotic Bacterial Strains

The evaluation of the hemolytic potential of the postbiotics and probiotics with the highest inhibitory activity was performed as described by Mendonça et al. (2021) [25]. Fresh canine blood samples were collected in EDTA tubes from healthy donors, after the owners gave their consent. To isolate canine red blood cells (cRBCs), the samples were centrifuged at 1000 g for 10 min at 4 °C, washed three times, and resuspended in sterile PBS buffer (pH 7.4) to obtain a 0.5% (*v*/*v*) cRBC suspension. Then, the cRBC suspension was separately mixed with each of the biotics in a sterile, round-bottomed 96-well microtiter polypropylene plate and incubated for 4 and 24 h at room temperature under gentle swirling. The final biotic concentration used was 50%, 12.5%, or 5% depending on the biotic, and the final cRBC concentration was 0.25% (*v*/*v*). After incubation, the plate was centrifuged at 1000 g for 5 min at 4 °C, and the supernatants were transferred to a new sterile, clear, flat-bottomed 96-well microtiter polystyrene plate. The hemoglobin release from lysed cells was quantified by measuring the absorbance at 415 nm using a microtiter plate reader (BMG Labtech, FLUOstar OPTIMA, Ortenberg, Germany). Samples incubated with PBS were used as the negative control and samples incubated with Triton X-100 at 1% (*v*/*v* in H_2_O) as the positive control.

Hemolytic activity (%) was determined using the following equation:

Hemolytic activity (%) = [(Abs_treated_ − Abs_untreated_)/(Abs_Triton_ − Abs_untreated_)] × 100

where Abs_treated_ is the absorbance of the biotic-treated cells, Abs_untreated_ is the absorbance of samples incubated with PBS, and Abs_Triton_ is the absorbance of samples incubated with Triton X-100. Experiments were conducted in triplicate in three independent assays.

### 2.13. Statistical Analysis

Data analysis was carried out using Microsoft Excel 2016^®^ and IBM SPSS Statistics 29.0 (IBM Corp., Armonk, NY, USA). Quantitative variables are expressed as mean values ± standard deviations. Statistical analysis was performed using Student’s *t*-test for comparisons between treatment durations and one-way ANOVA for comparisons among different postbiotic compounds and bacterial species. A significance level of 0.05 was considered.

## 3. Results

### 3.1. Preliminary Screening Results of Inhibitory Activity of Yeast-Derived Postbiotics and Probiotics

The screening of all biotics against five bacterial species, *N. zoodegmatis*, *C. canis*, *P. cangingivalis*, *P. canis*, and *E. faecalis*, was conducted using the spot-on-lawn method. Complete inhibition of *P. canis* was observed after exposure to YE-2 (50%), YE-3 (10%, 20%, and 50%), YE-5 (50%), YE-6 (50%), YE-7 (10%, 20%, and 50%), YCW-1 (10% and 20%), YCW-2 (10%), BC-05, and BC-37. For *N. zoodegmatis*, complete inhibition was achieved following exposure to YE-3 (20% and 50%) and the three probiotic bacterial strains. Additionally, all probiotic bacterial strains, YCW-1, and YCW-2 at all concentrations tested showed complete inhibitory potential against *C. canis*, while only partial inhibition of *P. cangingivalis* was noted following exposure to YE-3 at 50%. Finally, only the probiotic bacterial strains, i.e., BC-23, BC-05, and BC-37, presented inhibitory effects against *E. faecalis*. Detailed results, including the inhibitory halo diameters, are presented in Supplementary Files S1 and S2.

### 3.2. Results of Minimum Biofilm Inhibitory Concentration (MBIC) and Minimum Biofilm Eradication Concentration (MBEC)

The inhibitory potential of each postbiotic and probiotic is shown in Table 5. All biotics demonstrated some level of inhibition against the polymicrobial biofilm model used, with YE-4 and YCW-1 exhibiting the highest inhibition at the maximum concentrations tested. In terms of eradication potential, YCW-1 displayed anti-biofilm activity, achieving 17.28% eradication at the highest concentration tested (12.5%), and probiotic BC-05 could eradicate 4.23% of the biofilm at the highest concentration tested (10^9^ CFU/mL).

**Figure 1:**
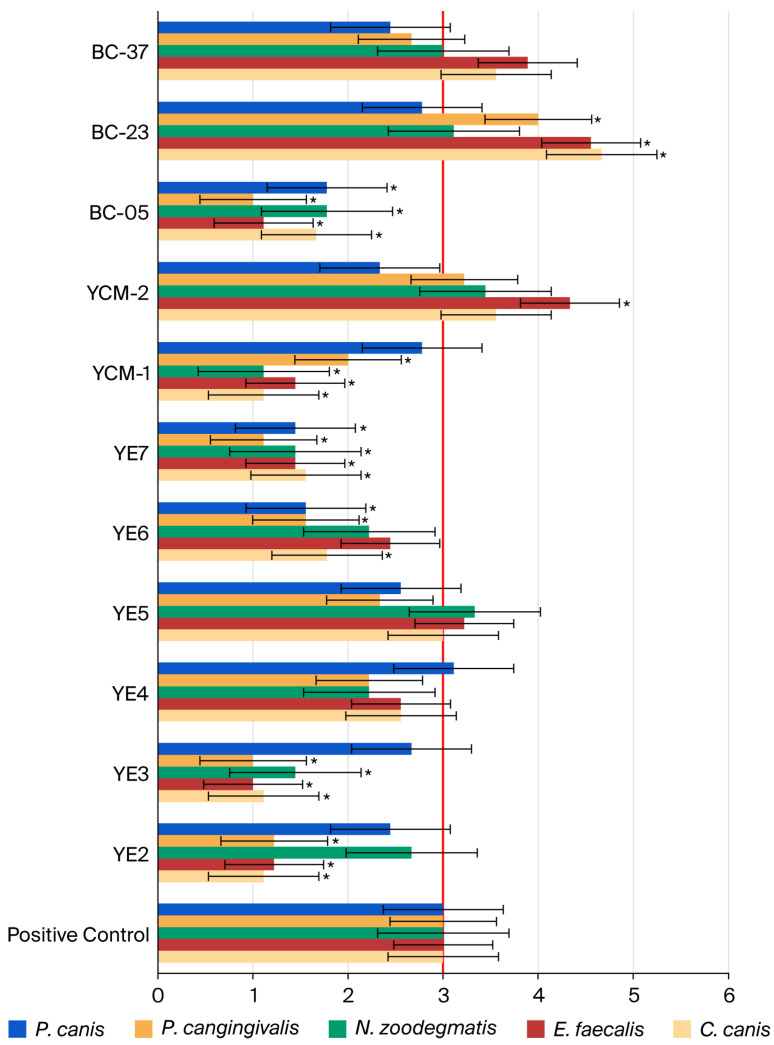
Representation of the FISH detection results of individual bacterial species in the biofilm following exposure to the highest concentration of postbiotics or probiotics, compared with the positive control (untreated polymicrobial biofilm). Results are presented using a scoring system ranging from 1 to 5. The red line represents the bacterial detection levels of the untreated polymicrobial biofilm. * *p*-Value < 0.05, compared with the positive control.

**Figure 2:**
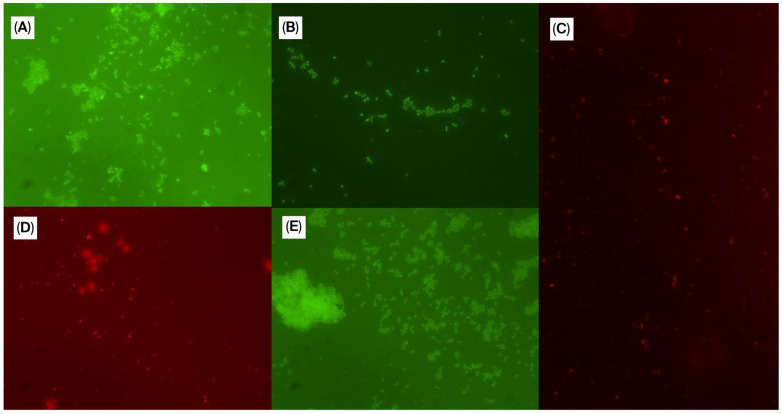
Detection of individual bacterial species in the polymicrobial biofilm based on FISH without exposure to postbiotics or probiotics. (**A**) *Enterococcus faecalis*; (**B**) *Corynebacterium canis*; (**C**) *Neisseria zoodegmatis*; (**D***) Peptostreptococcus canis*; (**E**) *Porphyromonas cangingivalis*.

Figure 1 summarizes the antimicrobial activity of each biotic against each of the bacterial species present in the polymicrobial biofilm according to the FISH evaluation, in which the positive control (polymicrobial biofilm without any treatment) was used as the basis for comparison (Figure 2). The majority of the postbiotic compounds promoted a reduction in all bacterial species, with the exception of YCW-2 against *C. canis*, *E. faecalis*, *N. zoodegmatis*, and *P. cangingivalis*; YE-5 against *E. faecalis* and *N. zoodegmatis*; and YE-4 against *P. canis*.

Among the probiotic bacterial strains, BC-05 was the most promising, as it led to a reduction in all bacterial species of the biofilm, while probiotic strains BC-23 and BC-37 showed minor activity against *P. canis*.

According to the statistical analysis, significant differences (*p* < 0.05) were observed between the evaluated postbiotic compounds and bacterial species. Specifically, YE-2, YE-3, YE-6, YE-7, YCW-1, YCW-2, BC-05, and BC-23 showed differences when compared with the positive control, as indicated in Figure 1. Among them, BC-05 and YE-7 induced a statistically significant reduction in all bacterial species relative to the positive control.

### 3.3. Inhibition and Eradication Potential of Dual Combinations of Yeast-Derived Postbiotics and a Probiotic Bacterial Strain

Based on the results obtained, four promising yeast-derived postbiotics and one probiotic bacterial strain were selected for further evaluation: YE-3, YE-7, YCW-1, YCW-2, and BC-05, respectively. The results of the antimicrobial activity of the postbiotic–probiotic combinations are presented in Table 6. The combination of YE-3 plus YE-7 showed the highest inhibitory activity against the polymicrobial biofilm. No statistically significant differences (*p* > 0.05) were observed among the dual combinations.

### 3.4. Determination of Inhibitory Activity of Yeast-Derived Postbiotics and Probiotic Bacterial Strain in the Presence of Canine Saliva

The inhibitory activity of the four selected yeast-derived postbiotics (YE-3, YE-7, YCW-1, and YCW-2) and of probiotic bacterial strain BC-05 against each of the bacterial species was evaluated in the presence of canine saliva with the spot-on-lawn method. YCW-1 (8.33 mm ± 0.76), YCW-2 (5 mm ± 0.87), and BC-05 (9.33 mm ± 0.58) produced a complete inhibition halo in the *C. canis* cultures, with YCW-1 (3 mm ± 0.5) and BC-05 (11.33 ± 1.15) also producing a complete inhibition halo in the *P. canis* cultures. None of the other bacterial species were inhibited by the other postbiotic compounds.

### 3.5. Determination of Hemolytic Potential of Yeast-Derived Postbiotics and Probiotic Bacterial Strain

Finally, we assessed the hemolytic activity of the four postbiotic compounds and the probiotic and found that all demonstrated hemolytic activity, with BC-05 presenting the lowest level. The results for each biotic are presented in Table 7. No statistical differences (*p* > 0.05) were observed regarding the exposure treatment time (4 vs. 24 h), nor among the biotics after 4 h of exposure. However, after 24 h of exposure, BCC05 and YCW-1 showed the lowest hemolytic activity, which was statistically significantly different (*p* < 0.05) from that of the other compounds.

## 4. Discussion

PD affects over 80% of adult dogs and is primarily driven by polymicrobial biofilm formed on the tooth surface, along with a local inflammatory response [1,2]. Most strategies aiming at improving canine oral health and preventing PD rely on the mechanical removal of dental plaque [10]. In humans, probiotic and postbiotic compounds have been shown to be able to inhibit periodontal pathogens, modulate inflammatory cytokines, and improve clinical dental indices [9,11,26,27]. Specifically, in dogs, recent evidence has shown that probiotics and postbiotics can also be useful in PD control. You et al. (2022) described some probiotics as promising compounds to support oral health without systemic adverse effects, and Florit-Ruiz et al. (2025) showed that a postbiotic compound has the potential to support oral health in dogs by modifying the supragingival plaque, inhibiting some early colonizers such as *N. zoodegmatis*, and reducing dental plaque formation [10,28]. Therefore, probiotics and postbiotics may represent a pioneering approach to preventing PD in dogs.

In this study, eleven probiotics and postbiotics were tested for antimicrobial potential against periodontal pathogens. All the evaluated biotics showed biofilm inhibitory activity, with YE-4 and YCW-1 achieving the highest inhibition at the maximum concentrations evaluated, followed by YE-2 and BC-05. In addition, a FISH technique was used to evaluate the specific antimicrobial activity against the bacterial species constituting the polymicrobial biofilm model [14]. This method enabled us to target each bacterial species of the biofilm and perform a semi-quantitative evaluation of its amount, using an innovative scale based on biofilm values ranging from 1 to 5. This scale was defined based on the positive control (biofilm without any treatment), represented by the value of 3; higher amounts of bacteria were classified with a score of 4 or 5 and lower bacterial levels with 1 or 2. Most of the postbiotic compounds promoted a reduction in all bacterial species, with the exception of YCW-2, YE-5, and YE-4. On the other hand, among the probiotic strains, only BC-05 was able to reduce all bacterial species present in the biofilm. Statistically significant reductions in all bacterial species were observed with BC-05 and YE-7.

Probiotics and postbiotic compounds may differ in their antimicrobial activity. You et al. (2022) reported similar findings when testing the antimicrobial activity of extracts from *Lactobacillus acidophilus* against several bacterial species [28].

Based on the evaluation results of the inhibitory activity of the individual biotics, four promising yeast-derived postbiotics and one probiotic bacterial strain were selected for further testing, namely, YE-3, YE-7, YCW-1, YCW-2, and BC-05, respectively. YCW-1 and YCW-2 were both included because they displayed distinct but complementary inhibition profiles: while YCW-1 showed broader inhibitory activity, YCW-2 was particularly effective against *P. canis*, a bacterium strongly linked to the progression of severe periodontal disease in dogs [29]. The antimicrobial potential of the selected compounds was investigated in dual combinations, and it was observed that the combination of postbiotics YE-3 plus YE-7 showed higher inhibitory activity against the polymicrobial biofilm, even higher than that displayed by the individual components. This highlights not only the differences among the biotics but also their potential variations in antimicrobial mechanisms. Notably, YE-7 and BC-05 exhibited antimicrobial activity in almost all combinations, with only one combination for each (YE-7 + YCM-1 and BC-05 + YE-3) showing no inhibitory potential. Overall, dual combinations tended to produce stronger inhibition than their components taken individually, suggesting additive or synergistic interactions between components. Similarly, other studies have focused on the activity of probiotic and postbiotic combinations, with researchers indicating that combinations exhibit higher antimicrobial activity than individual components [30,31].

The antimicrobial activity of probiotics and postbiotics has been attributed to several bioactive compounds, such as bacteriocins, organic acids, hydrogen peroxide, exopolysaccharides, and enzymes, which are able to disrupt bacterial membranes, inhibit biofilm formation, influence the immune response, and modulate microbial composition [26,31,32,33,34]. In addition, some of these compounds, such as the bioactive compound reuterin, obtained from the probiotic strain *Lactobacillus reuteri*, can have anti-inflammatory activity and promote the regeneration of periodontal tissues [35]. The antimicrobial effect of postbiotics may depend on several factors, such as the concentration of the postbiotic, the target bacteria, and the nature of the original compounds [34]. Therefore, future studies should investigate the biochemical composition of the biotics used in this study in order to identify the specific molecules responsible for the observed inhibitory activity of the individual biotics, as well as those contributing to the enhanced activity of the dual combinations.

After evaluating the antimicrobial potential of the individual compounds and their dual combinations and considering the objective of this study, i.e., identifying a solution for PD prevention, the stability of the antimicrobial activity of the most promising compounds was assessed in the presence of fresh canine saliva. Canine saliva has a complex composition, rich in proteins and several bioactive molecules, which can influence the activity of antimicrobial compounds [36]. For instance, Cunha et al. (2020) evaluated the effect of canine saliva on the antimicrobial activity of the bacteriocin nisin, finding it to be inhibitory [16]. In the present study, canine saliva similarly affected the antimicrobial activity of some of the tested compounds, depending on the evaluated bacterial species. Notably, postbiotic YCW-1 and probiotic BC-05, the most promising candidates, maintained their antimicrobial activity against *C. canis*, an early colonizer of dental plaque, and *P. canis*, a late colonizer associated with severe PD cases, in the presence of canine saliva [14,29]. These results reinforce the potential of these compounds for future research and clinical application in PD prevention strategies.

One of the initial approaches to assessing the toxic potential of compounds is the evaluation of their hemolytic activity. In this study, the hemolytic potential of the selected compounds was assessed by measuring the release of hemoglobin following 4 and 24 h of exposure. Previous studies have also investigated the hemolytic potential of probiotic strains, reporting variable results, with some strains showing no hemolytic activity and others demonstrating either complete or partial hemolysis [37,38,39]. This variability may be attributed to differences in the bacterial genetic and enzymatic machinery among probiotic strains. In our case, strain BC-05 displayed a hemolysis level of less than 10% and, based on other reports, can be considered to have no hemolytic potential [40], which makes it a promising compound for further studies. Regarding postbiotic compounds, the available literature remains limited, with most studies focusing on the anti-hemolytic or protective effects of these biotics rather than their potential to induce hemolysis. In this context, although our study identified a measurable hemolytic effect associated with the postbiotic compounds tested, further research is required to clarify their hemolytic potential and identify the specific bioactive compounds responsible for this activity.

This study evaluated the antimicrobial potential of eleven probiotic and postbiotic compounds against a model of canine dental plaque biofilm, revealing their potential for polymicrobial biofilm control. However, some limitations related to the use of in vitro methodologies must be acknowledged, as these methods do not fully replicate the in vivo environment. Moreover, the inherent complexity of biofilm structures may contribute to variability in their formation, which can result, in turn, in high standard deviations. Further studies are warranted to comprehensively characterize the biochemical composition of these compounds, evaluate their cytotoxicity, and ultimately perform in vivo experiments in dogs, where we could assess the ability of the most promising compounds or their combinations to reduce the risk of PD establishment or progression by measuring dental plaque coverage, periodontal indices, and indicators of adverse effects in canine subjects.

## 5. Conclusions

Dogs are frequently affected by periodontal disease (PD), which is one of the most widespread oral inflammatory conditions in veterinary medicine. In this study, the potential antimicrobial activity, as well as hemolytic potential, of eleven postbiotics and probiotics against periodontal bacteria in both planktonic and biofilm forms was evaluated. All tested biotics demonstrated biofilm inhibitory activity at their maximum concentrations against the evaluated polymicrobial PD biofilm; however, eradication was observed only with postbiotic YCW-1 and probiotic BC-05. When combined, YE-3 + YE-7 achieved a maximum inhibition rate of 71%. These findings reinforce the potential of these compounds to control bacteria associated with dental plaque, in line with previous studies that have also described postbiotics and probiotics as promising agents for oral health improvement. According to the safety evaluation of the compounds, probiotic BC-05 showed no hemolytic potential, making it the most promising candidate for further investigation. Future experiments should focus on the in vivo evaluation of these compounds for use in PD control in dogs.

## Figures and Tables

**Table 1:** Description of biotics, concentration of stock suspensions, and maximum final concentrations used in the assays in 96-well microplates.

Probiotic/Postbiotic	Stock/Suspension Concentration	Maximum Concentration per Well in a Microplate
YE-2	100%	50%
YE-3	100%	50%
YE-4	100%	50%
YE-5	100%	50%
YE-6	100%	50%
YE-7	100%	50%
YCW-1	25%	12.5%
YCW-2	10%	5%
BC-23	10^9^ CFU/mL	10^9^ CFU/mL
BC-05	10^9^ CFU/mL	10^9^ CFU/mL
BC-37	10^9^ CFU/mL	10^9^ CFU/mL

**Table 2:** Specific fluorescent probes used in the FISH protocol.

Bacterial Species	Sequence	Fluorophore	Reference
*Neisseria* sp.	5′-CGGGTGAGTAACATATCGG-3′	Rhodamine	[18]
*E. faecalis*	5′-TTATCCCCCTCTGATGGG-3′	Fluorescein	[19]
*Corynebacterium* sp.	5′-CCGGAATTTCACAGACGACG-3′	Fluorescein	[20]
*Porphyromonas* sp.	5′-TGTCAGTCGCAGTATGGCAA-3′	Fluorescein	[21]
*Peptostreptococcus* sp.	5′-TGCGCAAGCATGAAA-3′	Rhodamine	[22]

**Table 3:** Scoring system for biofilm assessment after Fluorescence In Situ Hybridization (FISH).

Score	Qualitative Assessment Level	Definition
1	Lower	Bacterial quantity clearly lower than the positive control
2	Slightly lower	Bacterial quantity lower than the positive control (up to approximately half of the bacterial quantity observed in the positive control)
3	Reference level	Bacterial quantity equivalent to the positive control
4	Slightly higher	Bacterial quantity higher than the positive control (up to approximately twice the reference level)
5	Higher	Bacterial quantity clearly higher than the positive control (more than twice the reference level)

**Table 4:** Selected dual combinations of yeast-derived postbiotics and probiotic bacterial strains and their components’ concentrations.

Combination	Concentrations of Components in Microplate Well
YE-3 + YE-7	25%/25%
YE-3 + YCW-1	25%/6.25%
YE-3 + YCW-2	25%/2.5%
YE-3 + BC-05	25%/10^9^ CFU/mL
YE-7 + YCW-1	25%/6.25%
YE-7 + YCW-2	25%/2.5%
YE-7 + BC-05	25%/10^9^ CFU/mL
YCW-1 + YCW-2	6.25%/2.5%
YCW-1 + BC-05	6.25%/10^9^ CFU/mL
YCW-2 + BC-05	2.5%/10^9^ CFU/mL

**Table 5:** Results of mean inhibition potential (in percentage) of yeast-derived postbiotics and probiotic bacterial strains against polymicrobial biofilm according to crystal violet method.

Compound	Concentrations (%) of Postbiotic Compounds									
	50.00%	25.00%	12.50%	6.25%	3.13%	1.56%	0.78%	0.39%	0.20%	0.10%
YE-2	16.86	15.34	7.68	3.99	2.13	0.00	0.00	0.00	0.00	0.00
YE-3	6.01	0.00	0.00	0.00	0.00	0.00	0.00	0.00	0.00	0.00
YE-4	21.66	15.79	17.49	13.90	6.37	2.47	0.00	0.00	0.00	0.00
YE-5	14.27	13.60	12.15	9.20	0.00	0.00	0.00	0.00	0.00	0.00
YE-6	22.55	20.89	16.52	14.24	4.83	0.00	0.00	0.00	0.00	0.00
YE-7	13.63	14.62	12.76	15.02	8.07	3.78	0.00	0.00	0.00	0.00
	**Concentrations (%) of Postbiotic Compound**									
	**12.50%**	**6.25%**	**3.13%**	**1.56%**	**0.78%**	**0.39%**	**0.20%**	**0.10%**	**0.05%**	**0.02%**
YCW-1	19.45	18.62	14.29	10.26	4.36	3.53	2.82	3.75	4.09	6.11
	**Concentrations (%) of Postbiotic Compound**									
	**5.00%**	**2.50%**	**1.25%**	**0.63%**	**0.31%**	**0.16%**	**0.08%**	**0.04%**	**0.02%**	**0.01%**
YCW-2	12.91	10.03	6.91	3.47	2.30	0.00	0.00	0.00	0.00	0.00
	**Concentrations (CFU/mL) of Probiotic Bacterial Strains**									
	**10^9^**	**10^8^**	**10^7^**	**10^6^**	**10^5^**	**10^4^**	**10^3^**	**10^2^**	**10^1^**	**10^0^**
BC-23	6.47	2.35	0	0	0	0	0	0	0	0
BC-05	16.11	2.70	0	0	0	0	0	0	0	0
BC-37	13.69	4.69	1.39	3.40	2.37	0.37	1.01	0	0	0

**Table 6:** Results of the inhibition and eradication activity of the yeast-derived postbiotics and the probiotic bacterial strain evaluated in combination against the polymicrobial biofilm.

Postbiotic–Probiotic Components	% of Inhibition ± SD	% of Eradication ± SD
YE-3 + YE-7	71.57 ± 32.92	0.00
YE-3 + YCW-1	0.00	0.00
YE-3 + YCW-2	0.00	0.00
YE-3 + BC-05	0.00	0.00
YE-7 + YCW-1	0.00	0.00
YE-7 + YCW-2	41.11 ± 22.36	0.00
YE-7 + BC-05	33.14 ± 25.93	0.00
YCW-1 + YCW-2	0.00	0.00
YCW1 + BC-05	17.21 ± 34.37	0.00
YCW-2 + BC-05	25.03 ± 25.07	0.00

Legend: SD—standard deviation.

**Table 7:** Hemolytic activity (%) of the four yeast-derived postbiotics and the probiotic bacterial strain.

Postbiotic/Probiotic	Hemolytic Activity (%) ± SD	
	4 h	24 h
YE-3	65.13 ± 44.82	83.94 ± 19.89
YE-7	72.54 ± 10.88	73.29 ± 18.12
YCW-1	24.97 ± 18.64	31.34 ± 7.20
YCW-2	61.09 ± 40.80	62.19 ± 11.91
BC-05	6.80 ± 4.59	17.88 ± 13.21

Legend: SD—standard deviation.

## Data Availability

The data presented in this study is available in the article and Supplementary Files. Further inquiries can be directed to the corresponding author.

## Supplementary Materials

The following supporting information can be downloaded at: https://www.mdpi.com/article/10.3390/microorganisms13112472/s1, Supplementary File S1: Inhibition potential of extracts YE-2, YE-3, YE-4, YE-5, YE-6, YE-7, YCW-1, and YCW-2 of each of the bacterial species of the polymicrobial biofilm; Supplementary File S2: Representation of the preliminary inhibitory potential of the postbiotics and probiotics evaluated using the spot-on-lawn assay. (A) *Corynebacterium canis*; (B) and (C) *Neisseria zoodegmatis*; (D) *Enterococcus faecalis*.
