# Supplementary material for: Evaluation of Biofilm Inhibitory Activity of Probiotics and Postbiotics Using In Vitro Biofilm Model of Canine Periodontal Disease
Source: Microorganisms. 2025 Oct 29;13(11):2472. doi: 10.3390/microorganisms13112472 (PMC12654812; doi:10.3390/microorganisms13112472)
Supplement: Supplementary file 1 [file microorganisms-13-02472-s001.zip › Supplementary file S1.pdf]

Supplementary File S1: Inhibition potential of postbiotic yeast derivatives components YE-2, YE-3, YE-4, YE-5, YE-6, YE-7, YCW-1, and YCW-2, and probiotic bacterial strains BC-23, BC-05 and BC-37 by each bacterial species of the polymicrobial biofilm.

		***N. zoodegmatis* CCUG 52598T**			***C. canis* CCUG 58627T**		***E. faecalis***			***P. cangingivalis* DSMZ VPB 4874**			***P. canis* CCUG 57081**		
**Postbiotic/ concentration or strain**		**10^8 CFU/mL**	**10^7 CFU/mL**	**10^6 CFU/mL**	**10^8 CFU/mL**	**10^7 CFU/mL**	**10^8 CFU/mL**	**10^7 CFU/mL**	**10^6 CFU/mL**	**10^8 CFU/mL**	**10^7 CFU/mL**	**10^6 CFU/mL**	**10^8 CFU/mL**	**10^7 CFU/mL**	**10^6 CFU/mL**
**YE-2**	10%	N	I (5mm)	I (5mm)	I (9mm)	I (10mm)	N	N	N	N	N	N	N	N	I (4mm)
	20%	N	I (5mm)	I (5mm)	I (10mm)	I (10mm)	N	N	N	N	N	N	N	N	I (4mm)
	50%	I (5 mm)	I (10mm)	I (6mm)	I (10mm)	I (11mm)	N	N	N	N	N	I (6mm)	I (6mm)	I (5mm)	C (8mm)
**YE-3**	10%	I (5 mm)	I (5 mm)	I (5mm)	N	N	N	N	N	N	N	N	I (5mm)	I (5mm)	C (6mm)
	20%	I (5 mm)	I (6mm)	C (8mm)	N	N	N	N	N	N	N	N	I (5mm)	I (6mm)	C (6mm)
	50%	I (11 mm)	C (10mm)	C (12mm)	I (9mm)	I (7mm)	N	N	N	N	I (7mm)	I (8mm)	C (9mm)	C (10mm)	C (10mm)
**YE-4**	10%	N	N	N	N	N	N	N	N	N	N	N	N	N	N
	20%	N	N	N	N	N	N	N	N	N	N	N	N	N	N
	50%	N	N	N	N	N	N	N	N	N	N	N	N	N	N
**YE-5**	10%	N	N	N	N	N	N	N	N	N	N	N	N	N	N
	20%	N	N	N	N	N	N	N	N	N	N	N	I (4mm)	N	N
	50%	N	N	N	N	N	N	N	N	N	N	N	C (11mm)	I (5mm)	C (11mm)
**YE-6**	10%	N	N	N	N	N	N	N	N	N	N	N	N	N	N
	20%	N	N	N	N	N	N	N	N	N	N	N	N	N	I (5mm)
	50%	N	N	N	N	N	N	N	N	N	N	N	I (4mm)	I (4mm)	C (8mm)
	10%	N	N	N	N	N	N	N	N	N	N	N	C (11mm)	C (14mm)	C (12mm)
	20%	N	N	N	N	N	N	N	N	N	N	N	C (12mm)	C (16mm)	C (14mm)

**YE-7**	50%	I (5mm)	N	N	I (5mm)	N	N	N	N	N	N	N	C (14mm)	C (18mm)	C (15mm)
**YCW-1**	10%	I (16mm)	N	N	C (18mm)	C (20mm)	N	N	N	N	N	N	C (20mm	C (22mm)	C (26mm)
	20%	I (13mm)	N	N	C (15mm)	C (19mm)	N	N	N	N	N	N	C (18mm)	C (20mm)	C (24mm)
**YCW-2**	10%	N	N	N	C (15mm)	C (18mm)	N	N	N	N	N	N	C (25mm)	C (20mm)	C (24mm)
**BC-23**		C (26mm)	C (28mm)	C (26mm)	C (36mm)	C (36mm)	C (42mm)	N	N	N	N	N	N	N	N
**BC-05**		C (22mm)	C (26mm)	C (28mm)	C (36mm)	C (34mm)	C (36mm)	N	N	N	N	N	N	C (15mm)	C (13mm)
**BC-37**		C (20mm)	C (19mm)	C (22mm)	C (17mm)	C (17mm)	C (18mm)	N	N	N	N	N	N	C (25mm)	C (30mm)

Legend: N- negative; I - Incomplete inhibition (diameter inhibition zone); C- Complete inhibition (diameter inhibition zone). CFU – Colony forming unit.
