# Supplementary material for: Evaluation of Biofilm Inhibitory Activity of Probiotics and Postbiotics Using In Vitro Biofilm Model of Canine Periodontal Disease
Source: Microorganisms. 2025 Oct 29;13(11):2472. doi: 10.3390/microorganisms13112472 (PMC12654812; doi:10.3390/microorganisms13112472)
Supplement: Supplementary file 1 [file microorganisms-13-02472-s001.zip › Supplementary file S2.pdf]

**Supplementary File S2:** Representation of the preliminar inhibitory potential of the postbiotics and probiotic components evaluated using the spot-on lawn assay. A) *Corynebacterium canis*; B) and C) *Neisseria zoodegmatis*; D) *Enterococcus faecalis*

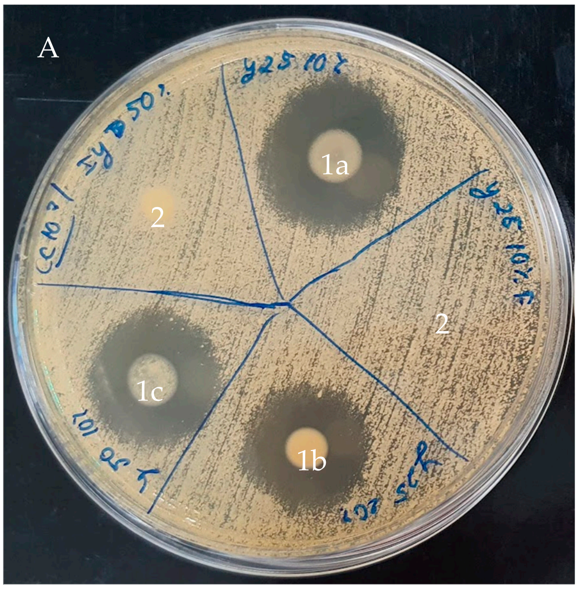


Legend: Spot-on-lawn assay using *C. canis* (lawn of $10^7$ CFU/mL). 1- complete inhibition observed with the components YCW-1 at 10% (1a) and 20% (1b), and YCW-2 at 10%(1c); 2 – no inhibition observed.

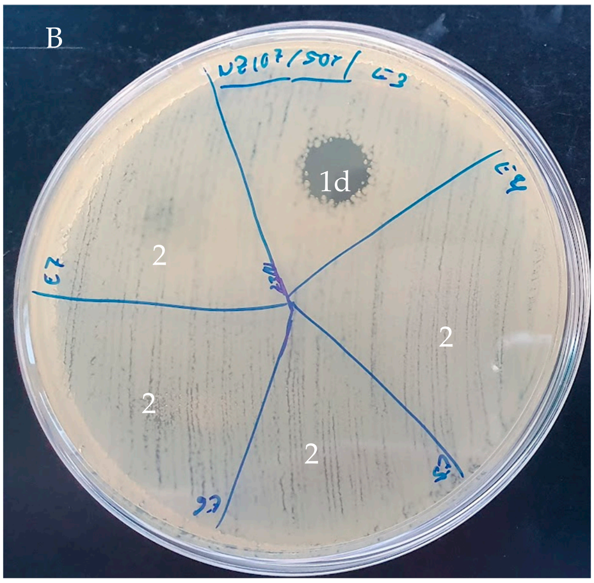


Legend: Spot-on-lawn assay using *N. zoodegmatis* (lawn of $10^7$ CFU/mL), 1- complete inhibition observed with the components EX-3 at 50% (1d); 2- no inhibition observed.

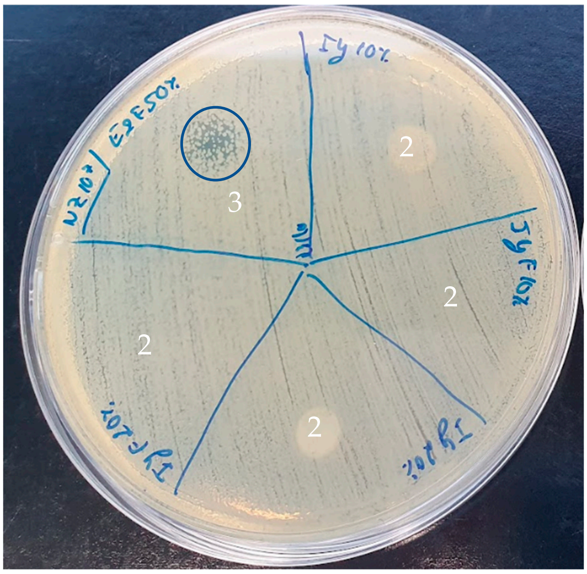


Legend: Spot-on-lawn assay using *N. zoodegmatis* (lawn of $10^7$ CFU/mL), 3-incomplete inhibition observed with the components YE-2 at 50%; 2- no inhibition observed.

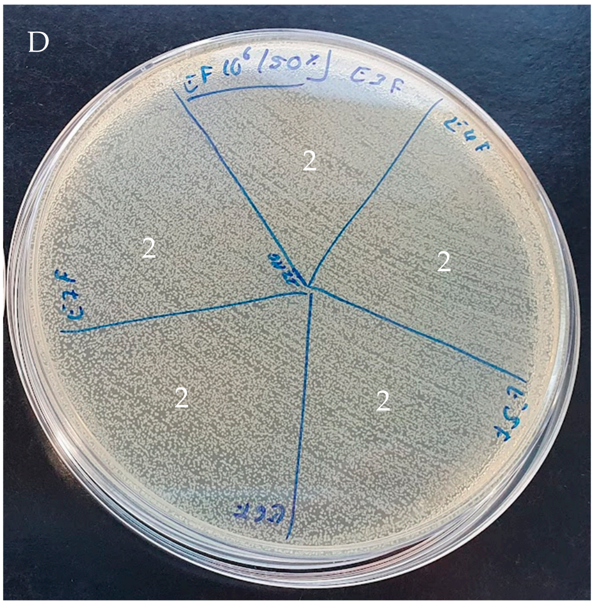


Legend: Spot-on-lawn assay using *E. faecalis* (lawn of $10^6$ CFU/mL); 2- no inhibition observed.
